# Phenotypic and molecular detection of the *bla*_KPC_ gene in clinical isolates from inpatients at hospitals in São Luis, MA, Brazil

**DOI:** 10.1186/s12879-016-2072-3

**Published:** 2016-12-07

**Authors:** Patricia Cristina Saldanha Ribeiro, Andrea Souza Monteiro, Sirlei Garcia Marques, Sílvio Gomes Monteiro, Valério Monteiro-Neto, Martina Márcia Melo Coqueiro, Ana Cláudia Garcia Marques, Rosimary de Jesus Gomes Turri, Simone Gonçalves Santos, Maria Rosa Quaresma Bomfim

**Affiliations:** 1 Programa de Pós-Graduação em Biologia Parasitária, Universidade CEUMA, Rua Josué Montello, No. 1, Renascença II, São Luís, Maranhão CEP 65075-120 Brazil; 2 Hospital Universitário da Universidade Federal do Maranhão, Rua Barão de Itapari, 227, Centro, São Luís, Maranhão Brazil; 3 Programa de Pós-Graduação em Saúde do Adulto e da Criança-UFMA, Universidade Federal do Maranhão, Av. dos Portugueses, 1966 - Bacanga, São Luis, Maranhão Brazil; 4 Departamento de Farmácia, Universidade Federal do Maranhão, Av. dos Portugueses, 1966 - Bacanga, São Luis, Maranhão Brazil; 5 Instituto de Ciências Biológicas, Universidade Federal de Minas Gerais, Av. Antonio Carlos, 6627, Pampulha, Belo Horizonte, MG Brazil

**Keywords:** *Klebsiella pneumoniae* carbapenemases (KPCs), *bla*_KPC_ gene variants, Multidrug-resistance (MDR), Enterobacterial repetitive intergenic consensus PCR (ERIC-PCR)

## Abstract

**Background:**

Bacteria that produce *Klebsiella pneumoniae* carbapenemases (KPCs) are resistant to broad-spectrum β-lactam antibiotics. The objective of this study was to phenotypically and genotypically characterize the antibiotic susceptibility to carbapenems of 297 isolates recovered from clinical samples obtained from inpatients at 16 hospitals in São Luis (Maranhão, Brazil).

**Methods:**

The study was conducted using phenotypic tests and molecular methods, including polymerase chain reaction (PCR), sequencing and enterobacterial repetitive intergenic consensus (ERIC)-PCR. The nonparametric chi-square test of independence was used to evaluate the associations between the bacterial *bla*_KPC_ gene and the modified Hodge test, and the chi-square adherence test was used to assess the frequency of carbapenemases and their association with the *bla*_KPC_ gene.

**Results:**

The most frequently isolated species were *Acinetobacter baumannii* (*n* = 128; 43.0%), *K. pneumoniae* (*n* = 75; 25.2%), and *Pseudomonas aeruginosa* (*n* = 42; 14.1%). Susceptibility assays showed that polymixin B was active against 89.3% of the bacterial isolates. The *Acinetobacter* spp. and *K. pneumoniae* strains were susceptible to amikacin and tigecycline, and *Pseudomonas* spp. were sensitive to gentamicin and amikacin. Among the 297 isolates, 100 (33.7%) were positive for the *bla*_KPC_ gene, including non-fermentative bacteria (*A. baumannii*) and *Enterobacteriaceae* species. Among the isolates positive for the *bla*_KPC_ gene, *K. pneumoniae* isolates had the highest positivity rate of 60.0%. The *bla*_KPC_ gene variants detected included KPC-2, which was found in all isolates belonging to species of the *Enterobacteriaceae* family. KPC-2 and KPC-3 were observed in *A. baumannii* isolates*.* Importantly, the *bla*_KPC_ gene was also detected in three *Raoultella* isolates and one isolate of the *Pantoea* genus. ERIC-PCR patterns showed a high level of genetic diversity among the bacterial isolates; it was capable of distinguishing 34 clones among 100 strains that were positive for *bla*_*KPC*_ and were circulating in 11 of the surveyed hospitals.

**Conclusions:**

The high frequency of the *bla*_*KPC*_ gene and the high degree of clonal diversity among microorganisms isolated from patients from different hospitals in São Luis suggest the need to improve the quality of health care to reduce the incidence of infections and the emergence of carbapenem resistance in these bacteria as well as other Gram-negative pathogens.

## Background

*Klebsiella pneumoniae* carbapenemases (KPCs) are β-lactamases commonly produced by *bla*_KPC_ gene-harboring bacteria of the *Enterobacteriaceae* family, such as *Klebsiella pneumoniae*, *Escherichia coli* and *Enterobacter* spp. [1, 2]. These enzymes hydrolyze a wide spectrum of β-lactams, including penicillins, cephalosporins, and carbapenems [3]. The *bla*_KPC_ gene possesses different variants (KPC-2 to KPC-15) that are sporadically found in other species, such as non-fermenting bacteria [4–7].

The dispersion of bacteria carrying the *bla*_KPC_ gene in hospitals is a growing concern due to increased resistance to carbapenems, such as imipenem and meropenem, with significant increases observed in the minimum inhibitory concentrations (MICs) of these drugs for *K. pneumoniae* and other pathogens [8]. Furthermore, this dispersion appears to have increased globally, mainly in South America, with recent reports in Argentina and Brazil following the first report in Colombia in 2006 [9–11]. There is justifiable concern that new variants may arise, and horizontal transfer of the *bla*_KPC_ gene has been detected in other bacterial species commonly found in the hospital environment [12].

Most bacteria carrying the *bla*_KPC_ gene usually harbor other resistance genes, leading to resistance to multiple classes of antimicrobials, such as aminoglycosides and fluoroquinolones [13, 14]. When a carrier of a KPC variant gene evolves into a multidrug-resistant (MDR) microorganism, standard medical treatment has been found to be ineffective, and such infections are accompanied by high mortality rates [15].

In this study, we detected KPCs by phenotypic tests, such as the modified Hodge test (MHT), to confirm the presence of carbapenemase activity as recommended by the Clinical and Laboratory Standards Institute (CLSI), and the E-Test® (gradient method). Polymerase chain reaction (PCR) and genomic sequencing were used for the detection and identification of *bla*_KPC_ gene variants. Additionally, enterobacterial repetitive intergenic consensus (ERIC)-PCR was used to determine the genetic relatedness of the carbapenem-resistant isolates.

## Methods

### Biological samples

Clinical samples, including fluid abscess, rectal swab, blood, catheter tip, drenage secretion, nasal swab, surgical wound, tracheal secretion, and urine, were collected from June 2012 to July 2013, from inpatients at different hospitals that provide general assistance, emergency and outpatient care in São Luis city, Maranhão, Brazil. Of the 16 hospitals that provided samples, 14 (H01 to H10 and H13 to H16) were public hospitals in the São Luis public health system, and two (H11 and H12) were private healthcare facilities (Table 1). Biological samples were sent to the Laboratory Cedro (provider of services of Clinical Microbiology to all municipal and state hospitals fom the state of Maranhao). The following epidemiological data were provided by the hospitals: anatomical site of origin, hospital accomodation, and distribution by hospital sector.

**Table 1:** Distribution of Gram-negative bacilli obtained in São Luis, MA, from June 2012 to July 2013

Bacterial species																
Origin^a^	*A. baumannii*	*A. ursingii*	*P. aeruginosa*	*P. putida*	*P. fluorescens*	*E. aerogenes*	*E. cloacae*	*Pantoea* spp*.*	*P. mirabilis*	*E. coli*	*S. marcescens*	*K. pneumoniae*	*A. salmonicida*	*R. planticola*	*R. ornithinolytica*	Total (%)
H01	37	0	8	2	0	0	6	0	0	0	0	24	0	1	0	78 (26.3)
H02	15	0	1	0	1	0	1	0	0	0	0	0	0	0	0	18 (6.1)
H03	6	0	1	0	0	0	0	0	0	0	0	0	0	0	0	7 (2.4)
H04	3	0	1	0	0	0	0	0	0	0	0	0	0	0	0	4 (1.3)
H05	10	0	1	0	0	3	1	0	2	0	3	6	0	0	0	26 (8.8)
H06	3	1	8	1	0	1	0	0	0	0	1	0	0	0	0	15 (5.1)
H07	1	0	0	0	0	0	0	0	0	0	0	0	0	0	0	1 (0.3)
H08	0	0	0	0	0	0	0	0	0	0	0	1	0	0	0	1 (0.3)
H09	0	0	0	0	0	2	1	0	0	1	0	5	0	0	0	9 (3.0)
H10	44	0	16	1	0	1	3	0	0	1	0	25	0	1	1	93 (31.3)
H11	1	0	0	0	0	0	1	0	0	0	0	0	1	0	0	3 (1.0)
H12	5	0	5	1	0	2	4	2	1	0	1	14	0	0	0	35 (11.8)
H13	2	0	1	0	0	0	0	0	0	0	0	0	0	0	0	3 (1.0)
H14	0	0	0	0	0	0	1	0	0	0	0	0	0	0	0	1 (0.3)
H15	1	0	0	0	0	0	0	1	0	0	0	0	0	0	0	2 (0.7)
H16	0	0	0	0	0	0	1	0	0	0	0	0	0	0	0	1 (0.3)
Total	128	1	42	5	1	9	19	3	3	2	5	75	1	2	1	297 (100)

^a^Public hospitals (H01 to H10 and H13 to H16); Private hospitals (H11 and H12)

### Isolation and identification of bacterial strains

Biological specimens were processed in the Clinical Microbiology Section from Laboratory Cedro, where bacterial strains were isolated using MacConkey agar (Difco, Detroit, MI, USA), blood agar (bioMérieux), brain heart infusion (BHI) broth (Difco, Detroit, MI, USA) and CPS chromogenic agar (bioMérieux). Subsequently, the isolates were identified with Gram-negative (GN) cards, and their susceptibility profiles were determined using antimicrobial susceptibility (AST) test cards (N209 cards) by using the Vitek® 2 Compact system (bioMérieux, Marcy l’Etoile, France), according to the manufacturer’s instructions.

Of the isolates evaluated, 654 showed low sensitivity to carbapenems imipenem, meropenem or ertapenem and were frozen at −80 °C in BHI with 20% glycerol. We randomly selected 297 (45.4%) strains for this study. To ensure that there were no repeated bacterial isolates from the same patient, each selected bacterial strain had a code related to a patient’s medical record. At the Molecular Biology Laboratory of Microorganisms from University Ceuma, the bacterial strains were checked for purity. Confirmation of biochemical identification of all bacterial isolates was carried out by conventional tests.

### Disk diffusion antibiotic sensitivity testing

To confirm the antimicrobial susceptibility pattern of each clinical isolate, we performed the disk-diffusion (Kirby-Bauer) test on Mueller-Hinton agar with the following antimicrobial discs (Oxoid® Limited, Basingstoke, UK): AMI, amikacin (30 μg); AMP, ampicillin (10 μg); ASB, ampicillin/sulbactam (10/10 μg); ATM, aztreonam (30 μg); CFL, cephalothin (30 μg); CEF, cefepime (30 μg), CTX, cefotaxime (30 μg); CFO, cefoxitin (30 μg); CAZ, ceftazidime (30 μg), CIP, ciprofloxacin (5 μg); ERT, ertapenem (10 μg); GEN, gentamicin (10 μg); IMP, imipenem (10 μg); MER, meropenem (10 μg); and PTZ, piperacillin/tazobactam (100/10 μg); POL, Polymixin B (300 U); TIG, Tigecicline (15 μg). The diameters of zones of inhibition were measured, and results were interpreted in accordance with the criteria of Clinical and Laboratory Standards Institute [16].

### Determination of minimum inhibitory concentrations of carbapenems by E-Test®

The MICs were determined by the E-Test® (AB Biodisk, Solna, Sweden), according to the manufacturer’s instructions. The microorganism classifications included sensitive, intermediate and resistant to the antimicrobials, using breakpoints recommended by the document M100-S23 of the Clinical and Laboratory Standards Institute methods [16].

### Modified Hodge testing

The confirmation of carbapenemase activity was performed using a MHT as previously described [16]. The *K. pneumoniae* strain ATCC BAA-1706® was used as a negative control and *K. pneumoniae* ATCC BAA-1705® was used as a positive control. The presence of a distorted or clover leaf-shaped inhibition zone was interpreted as positive for carbapenemase-producing isolates, as recommended by the document M100-S24 of the Clinical and Laboratory Standards Institute methods [16].

### Molecular identification of isolated bacterial strains resistant to carbapenem

Total genomic DNA from the clinical isolates was obtained using a Wizard® Genomic DNA Purification Kit (Promega Corporation, Madison, USA), according to the manufacturer’s instructions. The concentration and purity of the extracted DNA were verified using a Nanodrop-ND1000 (Thermo Fisher Scientific, Waltham, MA, USA).

PCR assays were performed with primers for the *bla*_KPC_ family, Uni-KPC-F (5’-ATGTCACTGTATCGCCGTCT-3’) and Uni-KPC-R (5’-TTACTGCCCGTTGACGCCC-3’), as previously described [17]. This pair of primers amplifies the complete sequence of the *bla*_KPC_ gene (882 nucleotides). The PCR was performed in a Mastercycler thermocycler (Eppendorf, Foster City, California, USA) with AccuPrime DNA polymerase mix (1X buffer BII; 2 mM each dNTPs; 1.5 mM MgCl2; 200 mM Tris–HCl pH 8.4 and 1.5 U AccuPrime *Taq* DNA polymerase) (Invitrogen/Stratagene, La Jolla, CA, USA), 20 pmol of each primer, and 50 ng of genomic DNA to a final volume of 50 μl. The amplification reaction was performed using the following conditions: 94 °C for 3 min (initial denaturation) followed by 30 cycles of 94 °C for 1 min, 55 °C for 1 min and 72 °C for 1 min, and a final extension step at 72 °C for 5 min.

To verify the efficiency of the PCR and determine the sizes of the amplified DNA fragments, 8 μl of each PCR product was analyzed by electrophoresis on a 1.2% (wt/vol) agarose gel in Tris-acetate-EDTA buffer (TAE: 40 mM Tris-acetate and 1 mM EDTA). A 100 bp DNA ladder (Promega Corporation, Madison, USA) was included in each run. After electrophoresis, the agarose gels were stained with ethidium bromide (0.5 μg/ml) and photographed under ultraviolet light (UV) at 260 nm.

### Purification and sequencing of PCR products

To determine which *bla*_KPC_ variants were circulating in patients from hospitals in São Luis, Maranhão, the 882-bp amplified PCR products were purified using a commercial Wizard® SV Gel and PCR Clean-Up System Kit (both from Promega Corporation, Madison, USA), according to the manufacturer’s instructions. PCR purified products were sequenced by Myleus Biotechnology (Belo Horizonte, Minas Gerais, Brazil) using an ABI 3730XL DNA analyzer (Applied Biosystems, Carlsbad, CA, USA). Amplicons were bidirectionally sequenced at least three times; thus, each PCR product was sequenced six times.

The quality of the sequence electropherograms obtained during the sequencing process was analyzed with ChromasPro (http://www.technelysium.com.au/chromas.html) software. At least two sequences of each type of KPC were chosen from the database for alignments using MEGA 4.0 [18]. Similarities between the nucleotide sequences obtained were verified using BLASTN (https://blast.ncbi.nlm.nih.gov/Blast.cgi).

To identify the *bla*_KPC_ gene variants, all sequences were translated into amino acids using the software ExPASy [translate tool (http://web.expasy.org/translate/) in the six reading frames]. The correct translation was chosen based on the available data in GenBank. Deduced amino acid sequences were compared with KPC protein sequences from GenBank using BLASTX. Similarity values for amino acid sequences ranged from 99 to 100%, indicating a highly conserved region.

### Identification of *Acinetobacter baumannii* by detection of the *bla*_OXA-51-like_ carbapenemase gene

PCR assays for *bla*_OXA-51_-like carbapenemase, a gene intrinsic to this species, were performed for all isolates identified by biochemical tests as *Acinetobacter*. As a positive control, we used a strain of *A. baumannii* ATCC19606. Amplifications were carried out with specific primers previously described [19], and the PCR products were analysed by electrophoresis on a 2.0% (wt/vol) agarose gel containing 0.5 μg/ml ethidium bromide.

### ERIC-PCR amplifications and profile analysis

ERIC-PCR amplifications were used for molecular typing of all isolates analyzed in this study. PCR assays were performed with 100 ng of genomic bacterial DNA, 10 pmol of each ERIC-1R (5’-ATGTAAGCTCCTGGGGATTCAC-3’) and ERIC-2 (5’-AAGTAAGTGACTGGGGTGAGCG-3’) primer (Invitrogen, Carlsbad, CA, USA), as previously described [20], plus 12.5 μl of Master Mix (Promega Corporation, Madison, USA) and ultra-pure water to bring the final reaction volume to 25 μl. The PCR conditions included an initial denaturation at 94 °C for 3 min followed by 35 cycles of 94 °C for 30 s, 52 °C for 1 min, and 72 °C for 2 min, with a final extension at 72 °C for 6 min. PCR amplicons were resolved by electrophoresis on a 2% (w/v) agarose gel in acetate-EDTA buffer. A 100 bp DNA ladder (Promega Corporation, Madison, USA) was included in each run. After electrophoresis, the gels were stained with ethidium bromide (0.5 μg/ml), and photographed under UV at 260 nm.

ERIC-PCR profiles were scored by fragment size with the LabImage-1D gel analysis software, Version 3.2 (1D: V6.2. Available in: http://www.kapelanbio.com/). Amplified fragments were scored as absent (0) or present (1) to construct a dendrogram. ERIC-PCR genotype dendrograms were constructed based on the average similarity of the matrix using the unweighted pair group method with arithmetic mean and the DICE similarity coefficient using NTSYS-pc version 2.1, Exeter Software (New York, NY, USA). The nearest neighbor-joining clustering method was used to show relationships between similar groups.

### Statistical analysis

To evaluate the associations between the *bla*_KPC_ gene variants and the modified Hodge test, the nonparametric chi-square test of independence was used. To assess the frequency of carbapenemases and their association with the presence of the *bla*_KPC_ gene, we used the chi-square adherence test. The data were considered statistically significant at *p* < 0.05.

## Results

### Identification of bacterial species and susceptibility tests

In the present study, susceptibility testing was performed on 297 bacterial strains (*Enterobacteriaceae* and non-fermentative Gram*-*negative bacilli) isolated from June 2012 to July 2013, which showed decreased sensitivity to imipenem, ertapenem or meropenem. These bacterial strains belonged to the genera *Acinetobacter, Klebsiella*, *Enterobacter*, *Pseudomonas*, *Serratia*, *Pantoea*, *Proteus*, *Escherichia*, *Raoultella*, and *Aeromonas*. Most of the strains were identified as *A. baumannii* (*n* = 128, 43.1%), *K. pneumoniae* (*n* = 75, 25.2%), and *P. aeruginosa* (*n* = 42, 14.1%).

The susceptibility testing and minimum inhibitory concentrations of bacterial species indicated that members of the *Enterobacteriaceae* family had elevated MICs for most antimicrobial agents tested, except for Polymixin. For meropenem and imipenem, a MIC of ≥ 32 μg/ml was detected for all strains of *K. pneumoniae*. Simultaneous drug resistance to fluoroquinolones, aminoglycosides and other β-lactam antibiotics was observed for 66 bacterial isolates (Table 2). In addition, low percentages of resistance to amikacin were observed for most strains. *K. pneumoniae* isolates were susceptible to amikacin and tigecycline; *Raoultella planticola* isolates showed sensitivity only to amikacin; and *R. ornithinolytica* showed sensitivity to amikacin, gentamicin, and tigecycline. *S. marcescens* strains, which are intrinsically resistant to Polymixin B, were highly sensitive to fluoroquinolone (ciprofloxacin) (Table 2).

**Table 2:** Antibiotic resistance profiles and minimal inhibitory concentrations for fermentative bacilli species

	fermentative gram-negative bacilli																			
Antibiotics	*K. pneumoniae*		*E. cloacae*		*E. aerogenes*		*S. marcescens*		*Pantoea* spp*.*		*P. mirabilis*		*R. planticola*		*R. ornithinolytica*		*E.coli*		*A. salmonicida* ^a^	
	%	MIC	%	MIC	%	MIC	%	MIC	%	MIC	%	MIC	%	MIC	%	MIC	%	MIC	%	MIC
amikacin	12	≥64	5.3	≥64	33.3	≥64	80.0	≥64	0.0	16	33.3	≥64	0.0	16	0.0	4	0.0	≤2	0.0	4
ampicillin	100	≥32	100	≥32	100	≥32	100	≥32	100	≥32	100	≥32	100	≥32	100	≥32	100	≥32	100	≥32
amp/sulbac^b^	100	≥32	100	≥32	100	≥32	100	≥32	100	≥32	100	≥32	100	≥32	100	≥32	100	≥32	100	≥32
aztreonam	100	≥64	100	≥64	100	≥64	100	≥64	100	≥64	100	≥64	100	≥64	100	≥64	100	≥64	100	≥64
cephalothin	100	≥64	100	≥64	100	≥64	100	≥64	100	≥64	100	≥64	100	≥64	100	≥64	100	≥64	100	≥64
cefepime	100	≥64	100	≥64	100	≥64	100	≥64	100	≥64	100	≥64	100	≥64	100	≥64	100	≥64	100	≥64
cefotaxime	100	≥64	100	≥64	100	≥64	100	≥64	100	≥64	100	≥64	100	≥64	100	≥64	100	≥64	100	≥64
cefoxitin	100	≥64	100	≥64	100	≥64	100	≥64	100	≥64	100	≥64	100	≥64	100	≥64	100	≥64	100	≥64
ceftazidime	100	≥64	100	≥64	100	≥64	100	≥64	100	≥64	100	≥64	100	≥64	100	≥64	100	≥64	100	≥64
ciprofloxacin	93.3	≥4	89.5	≥4	55.6	≥4	0.0	≤0.25	66.7	≥4	66.7	≥4	100	≥4	100	≥4	50.0	≥4	0.0	≤0.5
polymyxin B	10.7	64	0	≤0.5	0.0	≤0.5	100	≥16	0.0	≤0.5	100	≥16	100	≥16	100	≥16	0.0	≤0.5	NT^d^	NT^d^
gentamicin	52	≥16	68.4	≥16	11.1	≥16	40.0	≥16	33.3	≥16	66.7	≥16	100	≥16	0.0	≤1	50.0	≥16	0.0	≤1
imipenem	85.3	≥32	42.1	≥32	88.9	≥32	80.0	≥16	33.3	≥16	100	≥32	100	≥16	100	≥16	0.0	≤1	100	≥16
meropenem	84	≥32	42.1	≥32	88.9	≥32	80.0	≥16	33.3	≥16	66.7	≥32	50.0	≥16	100	≥16	0.0	≤0.25	100	≥16
ertapenem	100	≥32	100	≥32	88.9	≥32	100	≥8	100	≥8	100	≥32	100	≥8	100	≥8	100	≥8	100	≥8
piper/tazob^c^	100	≥128	94.7	≥128	88.9	≥128	100	≥128	100	≥128	66.7	≥128	100	≥128	100	≥128	100	≥128	0.0	16
tigecycline	21.3	≥8	52.6	≥8	22.2	≥8	40.0	≥8	NT^d^	NT^d^	100	≥8	100	4	0.0	2	0.0	≤0.5	NT^d^	NT^d^
	75		19		9		5		3		3		2		1		2		1	

^a^ Family Aeromonadaceae

^b^ amp/sulbac = ampicillin*/*sulbactam

^c^ piper/tazob = piperacillin-tazobactam

^d^ NT = non-tested

The multidrug-resistant (MDR) non-fermentative isolates were susceptible to Polymixin B, with an MIC of ≤ 0.5 μg/ml for all isolates. *A. baumannii* and *P. aeruginosa* had MICs of ≥16 μg/ml for meropenem and imipenem. In addition, MICs of ≥ 64 μg/ml for amikacin and 4 μg/ml for tigecycline were found in *Acinetobacter* spp. isolates, and MICs of ≥ 64 μg/ml for amikacin and ≥ 16 μg/ml for gentamicin were found in *Pseudomonas* spp. isolates; the antimicrobial resistance profiles of the other isolates showed values above 50% (Table 3).

**Table 3:** Antibiotic resistance profiles and minimal inhibitory concentrations for non-fermentative bacilli species

	non-fermentative gram-negative bacilli									
Antibiotics	*Acinetobacter baumannii*		*Acinetobacter ursingii*		*Pseudomonas aeruginosa*		*Pseudomonas fluorescens*		*Pseudomonas putida*	
	%	MIC	%	MIC	%	MIC	%	MIC	%	MIC
amikacin	22.7	≥64	0.0	4	28.6	≥64	0.0	≤2	0.0	16
ampicillin	100	≥32	100	≥32	100	≥32	100	≥32	100	≥32
amp/sulbac^a^	72.7	≥32	100	≥32	100	≥32	100	≥32	100	≥32
aztreonam	100	≥64	100	≥64	NT^c^	NT^c^	NT^c^	NT^c^	NT^c^	NT^c^
cephalothin	100	≥64	100	≥64	NT^c^	NT^c^	100	≥64	100	≥64
cefepime	97.7	≥64	100	≥64	64.3	≥64	100	≥64	60.0	≥64
cefotaxime	100	≥64	100	≥64	100	≥64	NT^c^	NT^c^	100	≥64
cefoxitin	100	≥64	100	≥64	100	≥64	100	≥64	100	≥64
ceftazidime	99.2	≥64	100	≥64	71.4	≥64	100	≥64	80.0	≥64
ciprofloxacin	96.9	≥4	0.0	≤0.5	69.0	≥4	100	≥4	40.0	≥4
polymyxin B	0.0	≤0.5	0.0	≤0.5	0.0	≤0.5	0.0	≤0.5	0.0	≤0.5
gentamicin	53.1	≥16	100	≥16	59.5	≥16	0.0	≤1	20.0	≥16
imipenem	100	≥16	100	≥16	100	≥16	100	≥16	100	≥16
meropenem	100	≥16	100	≥16	100	≥16	100	≥16	100	≥16
ertapenem	NT^c^	NT^c^	NT^c^	NT^c^	NT^c^	NT^c^	NT^c^	NT^c^	NT^c^	NT^c^
piper/tazob^b^	100	≥128	100	≥128	78.6	≥128	0.0	16	60.0	≥128
tigecycline	0.8	4	NT^c^	NT^c^	100	≥8	100	≥8	100	≥8
Total	128	1	42	1	5					

^a^amp/sulbac = ampicillin*/*sulbactam

^b^piper/tazob = piperacillin-tazobactam

^c^NT = non-tested

### Evaluation of the modified Hodge test (MHT)

The results of the MHT indicated a statistically significant association (*p* = 0.0001) with the assessed bacterial species. We observed that the microorganism with the highest positive test results was *K. pneumoniae* [60 (80%) of 75 isolates], followed by *Enterobacter cloacae* [7 (36.8%)]. (Table 4). There is no standardization of this test for non-fermenting bacteria (*Acinetobacter* spp. and *Pseudomonas* spp.) in the document M100-S24 of the Clinical and Laboratory Standards Institute methods [16]. Thus, for these microorganisms, the MHT was not performed.

**Table 4:** Association of the *bla*_*KPC*_ gene and the modified Hodge test with *Enterobacteriaceae* species

Enterobacteria	Total	*bla*_*KPC*_	MHT^a^	*bla*_*KPC*_ + MHT	*P*
		No. (%)	No. (%)	No. (%)	
*Enterobacter aerogenes*	9	6 (66.7)	7 (77.8)	5 (55.6)	0.0001
*Enterobacter cloacae*	19	6 (31.6)	7 (36.8)	4 (21.1)	
*Escherichia coli*	2	1 (50.0)	0 (0)	0	
*Klebsiella pneumoniae*	75	60 (80.0)	60 (80.0)	49 (65.3)	
*Pantoea* sp.	3	1 (33.3)	1 (33.3)	1 (33.3)	
*Proteus mirabilis*	3	0 (0)	1 (33.3)	0 (0)	
*Raoultella planticola*	2	2 (100)	2 (100)	2 (100)	
*Raoultella ornithinolytica*	1	1 (100)	1 (100)	1 (100)	
*Serratia marcescens*	5	2 (40.0)	3 (60.0)	2 (40.0)	
Total	119	79 (66.4)	82 (68.9)	64 (53.7)	

^a^MHT, modified Hodge test; *P* < 0.0001, Nonparametric chi-square test of independence

### Detection of the *bla*_*KPC*_ gene in bacterial isolates with decreased susceptibilities to carbapenems

We found a statistically significant correlation between the *bla*_KPC_ gene and bacterial species by the nonparametric chi-square test of independence (*p* < 0.05). The *bla*_KPC_ gene was detected in 100 (33.7%) of the 297 isolates evaluated in this study, including fermenting (*Enterobacteriaceae*) and non-fermenting (*Pseudomonas* and *Acinetobacter*) species. The *bla*_KPC_ gene was detected in *K. pneumoniae* (60 strains; 80.0%), *E. aerogenes* (6 strains; 66.7%), *E. coli* (1 strain; 50%), *Serratia marcescens* (2 strains; 40%), *E. cloacae* (6 strains; 31.6%), *Raoultella planticola* (2 strains; 100%), *R. ornithinolytica* (1 strain; 100%), and *Pantoea* sp. (1 strain; 33.3%) (Table 4). Of the 128 isolates of *A. baumannii*, 21 (16.4%) were positive for the *bla*_KPC_ gene.

The *bla*_KPC_ gene was not detected in the following bacterial species: *Acinetobacter ursingii, Aeromonas salmonicida*, *Proteus mirabilis*, *P. aeruginosa*, *P. fluorescens* and *P. putida*, although these microorganisms showed resistance or decreased susceptibility to carbapenems (imipenem, meropenem or ertapenem) in phenotypic tests.

We verified the high rate of *bla*_KPC_-positive *K. pneumoniae* among the isolates from four public hospitals: H05, 100.0% (6/6); H08, 100.0% (1/1); H09, 100.0% (5/5); H10, 80.0% (20/25) and H01, 75.0% (18/24). In addition, a high frequency of this gene was detected in H12, (71.4%, 10/14), a private hospital of high complexity in São Luis, MA.

Regarding the association between the modified Hodge test and the presence of the *bla*_KPC_ gene in species of the family *Enterobacteriaceae*, we observed positive results in both tests for 64 isolates (Table 4). Of importance, the concordance rate between the two tests was of 81% (64 out of 79 that were positive for the *bla*_KPC_ gene.

Of the non-glucose fermenting bacteria, only *A. baumannii* isolates carried the *bla*_KPC_ gene, and the largest number of isolates was recovered from three public hospitals in São Luis, MA: H02, 50.0% (5/10); H01, 19.0% (7/37) and H10, 18.1% (8/44).

The phenotypic profiles obtained by different methods (Vitek® 2, E-Test®, and the modified Hodge test) for all isolates that showed resistance to carbapenem antibiotics are shown in Table 5. Importantly, a high percentage of antimicrobial-resistant microorganisms harbored the *bla*_KPC_ gene.

**Table 5:** Frequency of antimicrobial-resistant microorganisms harboring *bla*KPC

	% antibiotic resistance strains with *blaKPC* gene^a^																	
Species	AMI	AMP	ASB	ATM	CFL	CEF	CTX	CFO	CAZ	CIP	POL	GEN	IMP	MER	ERT	PTZ	TIG	No. of *blaKPC +*
*Acinetobacter baumannii*	23.8	100	100	100	100	100	100	100	100	100	0.0	47.6	100	100	NT^b^	100	0.0	21
*Enterobacter aerogenes*	50.0	100	100	100	100	100	100	100	100	33.3	0.0	16.7	83.3	83.3	83.3	83.3	33.3	6
*Enterobacter cloacae*	20.0	100	100	100	100	100	100	100	100	100	0.0	60.0	100	100	100	100	40.0	6
*Escherichia coli*	0.0	100	100	100	100	100	100	100	100	100	0.0	100	0.0	0.0	100	100	NT^a^	1
*Klebsiella pneumoniae*	13.6	100	100	100	100	100	100	100	100	83.1	11.9	52.5	94.9	88.1	100	100	20.3	60
*Pantoea* spp*.*	0.0	100	100	100	100	100	100	100	100	100	0.0	100	100	100	100	100	NT^a^	1
*Raoultella planticola*	0.0	100	100	100	100	100	100	100	100	100	100	100	100	50.0	100	100	100	2
*Raoultella ornithinolytica*	0.0	100	100	100	100	100	100	100	100	100	100	0.0	100	100	100	100	0.0	1
*Serratia marcescens*	100	100	100	100	100	100	100	100	100	0.0	100	50.0	100	100	100	100	50.0	2

^a^AMI, amikacin (30 μg); AMP, ampicillin (10 μg); ASB, ampicillin/sulbactam (10/10 μg); ATM, aztreonam (30 μg); CFL, cephalothin (30 μg); CEF, cefepime (30 μg), CTX, cefotaxime (30 μg); CFO, cefoxitin (30 μg); CAZ, ceftazidime (30 μg), CIP, ciprofloxacin (5 μg); ERT, ertapenem (10 μg); GEN, gentamicin (10 μg); IMP, imipenem (10 μg); MER, meropenem (10 μg); and PTZ, piperacillin/tazobactam (100/10 μg); POL, Polymixin B (300 U); TIG, Tigecicline (15 μg)

^b^NT = non-tested

Statistical analyses using chi-square adherence tests revealed that the presence of the *bla*_KPC_ gene was significantly correlated with the distribution of the isolates at the site of infection (*p* < 0.0001). The highest frequency of this gene in the clinical isolates was 46.9% (30/64) in isolates from urine, followed by 39.5% (17/43) from blood, 39.4% (13/33) in nasal swabs, 33.3% (5/15) in rectal swabs and 21.4% in tracheal secretions (24/112). Specimens obtained from other anatomical sites showed low detection frequencies, which were probably due to the lack of quantitative uniformity among the materials collected during the study. Additionally, chi-square adherence tests indicated a correlation between the frequency of hospital accommodation and the presence of the *bla*_KPC_ gene (*p* < 0.0001).

The distribution of KPC-positive microorganisms by hospital sector revealed that 56 (32.7%) isolates were positive for the *bla*_KPC_ gene in 171 samples derived from the intensive care units (ICUs). By contrast, KPC-producing bacteria were found in several other units, except the pediatric ICU and the ward and labor rooms. The *bla*_KPC_ gene was also detected in bacterial isolates from patients admitted to sectors, such as the intermediate care unit, with four (20.0%) of the 20 cases from H10 and three (60.0%) of the five cases from H05, a hospital emergency room.

### Sequencing of the bla_KPC_ gene and amino acid sequence analysis

The *bla*_KPC_ gene was detected in 100 (33.7%) resistant bacterial isolates, and similarities among the sequences were assessed by performing alignments with nucleotide sequences from different bacteria. All sequences were deposited in GenBank with accession numbers KU695917 to KU696016. The similarity indices ranged from 99 to 100%, indicating that the sequenced region was highly conserved.

Nucleotide and amino acid differences between KPC enzymes (*bla*_KPC_ variants) were determined based on a study [6]. Amino acid analysis showed that type 2 (KPC-2) was the predominant variant for species from the *Enterobacteriaceae* family, including *R. planticola*, *R. ornithinolytica* and *Pantoea* spp. In addition, the KPC-2 and KPC-3 variants were predominant in *A. baumannii* strains.

### Detection of genes encoding OXA carbapenemase by Polymerase chain reaction

**Fig. 1:**
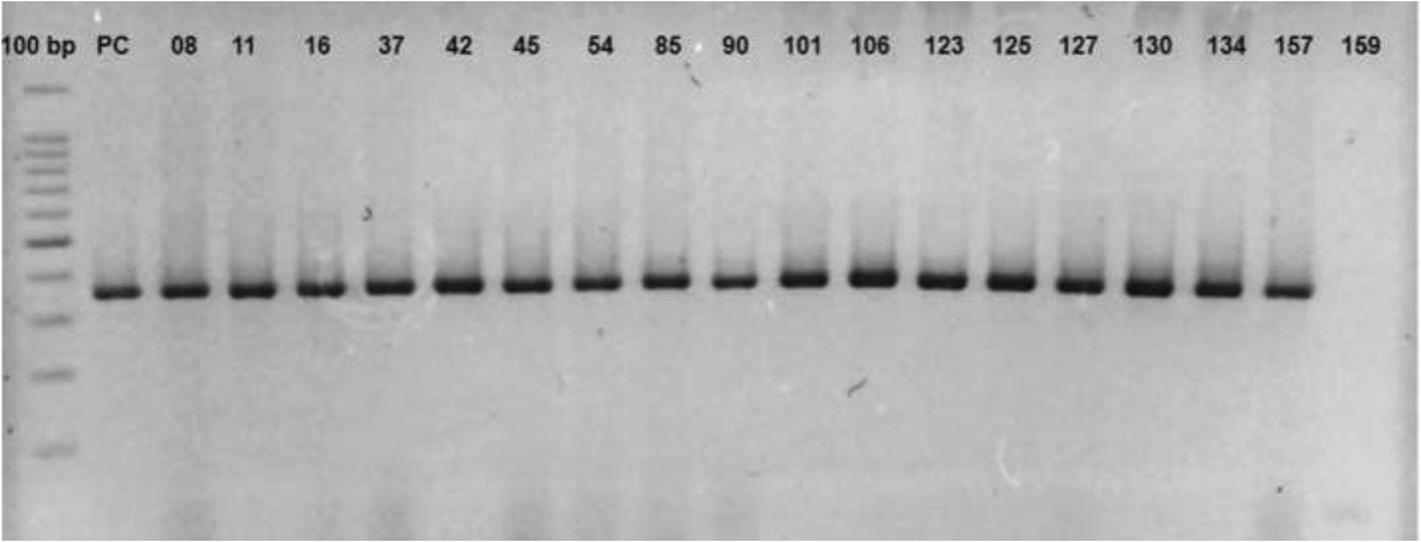
Agarose gel electrophoresis (2%) of the PCR products with primers specific for the *bla*_OXA-51_ gene in isolated evaluated in this study. Lane 1: Molecular pattern: 100 bp marker (Promega Corporation, Madison, USA); Lane 2: PC (positive control)/*Acinetobacter baumannii* ATCC19606; Lanes 3–19: Clinical isolates of *A. baumannii* harboring *bla*_OXA-51_ gene (353 bp), and Lane 20: Clinical isolates of *A. ursingii*

The primers specific for amplifying of the fragment of 353 bp for *bla*_OXA-51-like_ gene intrinsic to *A. baumannii* species showed that of the 129 isolates 128 harboured this gene. Only the isolate number 159, identified as *A. ursingii* not amplified with this pair of primer. Some representative results of amplification are shown in Fig. 1.

### DNA fingerprinting analysis by ERIC-PCR

PCR assays with ERIC primers showed heterogeneous genotypic patterns among the 297 isolates. Reproducible PCR patterns were observed for all isolates, with fragments that ranged from 70 to 1500 bp. Based on these polymorphic DNA fingerprinting patterns, ERIC-PCR analysis revealed clearly distinct DNA profiles from all the strains analyzed. Visual comparison of the banding patterns revealed 13 distinct ERIC profiles for 75 *K. pneumoniae* isolates, with molecular weights ranging from 80 to 950 bp. ERIC-PCR analysis for these isolates showed that 60 (80.0%) had identical profiles, whereas the other 15 isolates showed heterogeneous profiles.

**Fig. 2:**
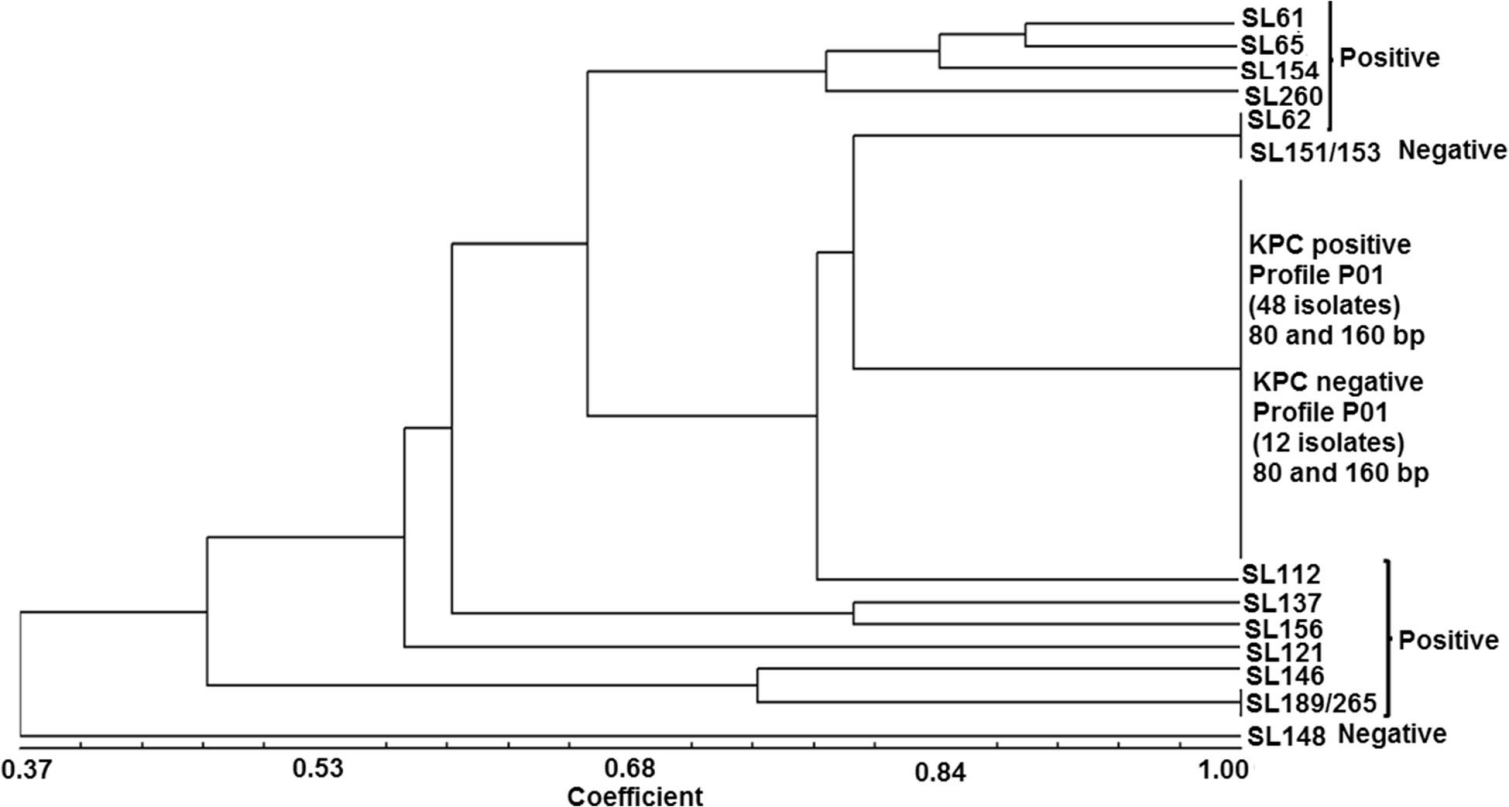
Dendrogram showing the genetic relatedness of 75 *Klebsiella pneumoniae* isolates. Cluster analysis of enterobacterial repetitive intergenic consensus PCR (ERIC-PCR) fingerprinting patterns using the DICE similarity coefficient and the UPGMA cluster method. The scale indicates the percentage of genetic similarity

ERIC-PCR analysis of the 60 *bla*_KPC_-positive *K. pneumoniae* strains showed an average of two to five fragments per isolate. Of these, the majority (48, 80.0%) showed a unique profile, with fragments of 80 and 160 bp (named P01 in Fig. 2). The other 12 isolates showed heterogeneous profiles, as follows: SL61 [80, 160, 280, 310, 500 bp]; SL62 [80, 160, 280 bp]; SL65 [80, 160, 280, 310 bp]; SL112 [80, 160, 750 bp]; SL121 [80, 100, 160, 200, 310 bp]; SL137 [80, 160, 280, 350, 550 bp]; SL146 [80, 160, 310, 350 bp]; SL154 [80, 160, 280, 310, 450 bp]; SL156 [80, 160, 200, 280, 550 bp]; SL189 and SL265 [160, 310, 350 bp] and SL260 [80, 160, 310 bp], as shown in Fig. 2.

Regarding the ERIC-PCR profiles of the 60 *bla*_KPC_-positive isolates, 20/25 (80.0%) positive isolates from H10 showed five different patterns: 16 isolates had identical profiles of 80 and 160 bp (P01, Fig. 2), and four isolates (SL62, SL112, SL154, SL260) had distinct profiles (Fig. 2). For H01, 18/24 (75.0%) positive isolates had four genetic ERIC-PCR profiles, with 15 of these isolates showing identical bands of 80 and 160 bp (P01, Fig. 2) and three isolates (SL137, SL146, SL156) with distinct genetic profiles (Fig. 2). For H05, 6/6 (100.0%) positive isolates had three different patterns, with fragments of 80 and 160 bp for four isolates (P01, Fig. 2) and distinct ERIC-PCR profiles for two isolates (SL121, SL265) (Fig. 2). For H09, all isolates [5/5 (100.0%)] showed a single profile of 80 and 160 bp (P01, Fig. 2). For H08, one isolate [1/1 (100.0%)] had fragments of 80 and 160 bp. For H12, 10/14 (71.4%) ERIC-PCR showed identical profiles (80 and 160 bp) for seven isolates (P01, Fig. 2), and SL61, SL65, and SL189 had distinct patterns (Fig. 2).

ERIC-PCR patterns for the 15 *bla*_KPC_-negative *K. pneumoniae* isolates showed amplicons of 80 and 160 bp for 12 isolates, and distinct DNA profiles with fragments ranging from 80 to 900 bp were amplified in three isolates: SL148 [160, 200, 300, 900 bp]; SL151; and SL153 [80, 160, 280 bp] (Fig. 2).

**Fig. 3:**
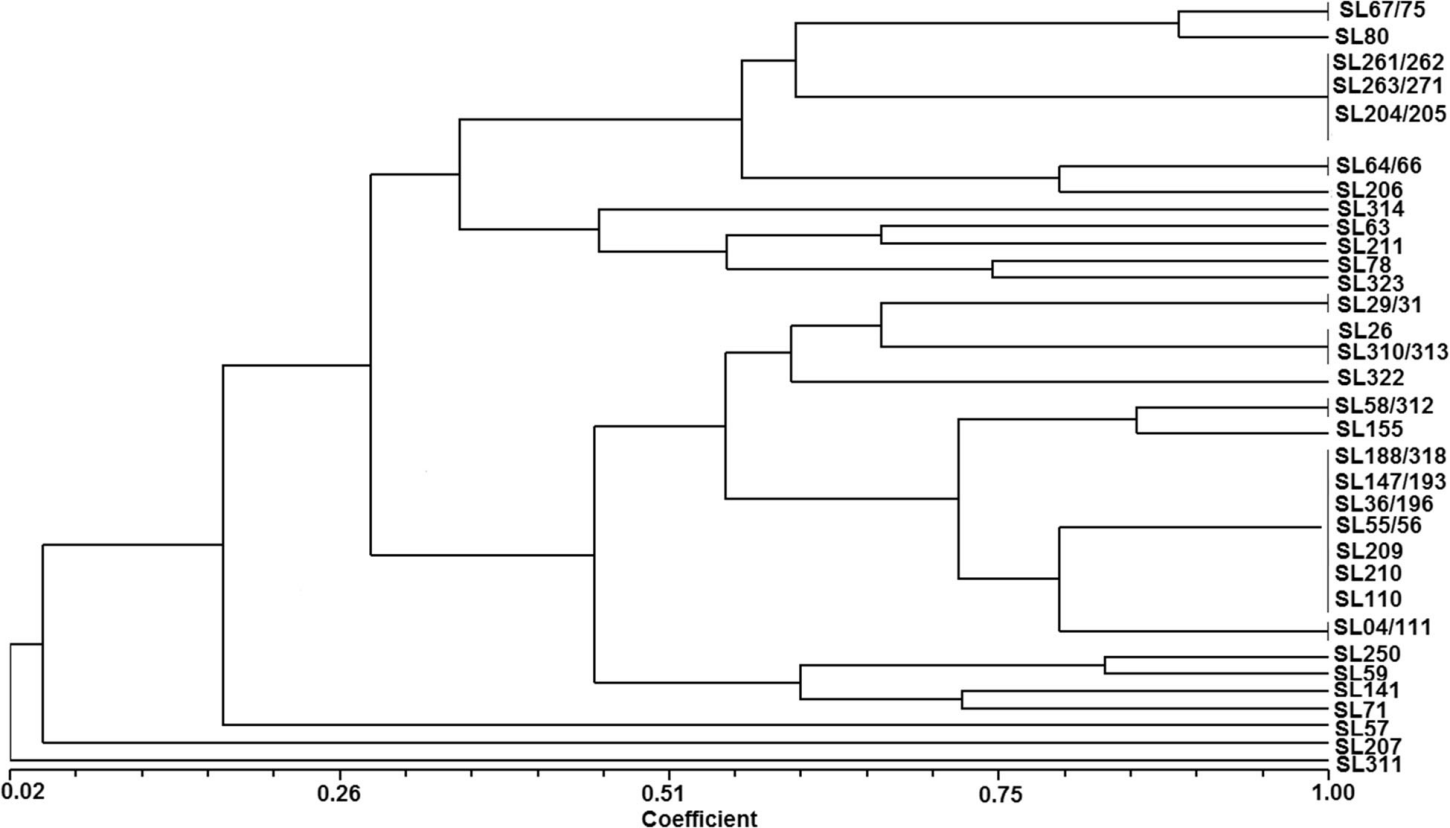
Dendrogram showing the genetic relatedness of 45 other isolates from the *Enterobacteriaceae* family. Cluster analysis of enterobacterial repetitive intergenic consensus PCR (ERIC-PCR) fingerprinting patterns using the DICE similarity coefficient and the UPGMA cluster method. The scale indicates the percentage of genetic similarity

ERIC-PCR patterns for the 19 *Enterobacter cloacae* isolates showed bands that ranged from 80 to 950 bp. The distinct profiles of 13 isolates that were negative for the *bla*_KPC_ gene were as follows: SL29 and SL31 [160, 450 bp]; SL58 and SL312 [80, 160, 310, 450 bp]; SL64 and SL66 [160, 310, 350, 450, 600 bp]; SL155 [80, 160, 450 bp]; SL188 and SL318 [80, 160 bp]; SL206 [160, 310, 350, 450, 480 bp]; SL262 and SL263 [310, 450 bp]; and SL311 [160, 600 bp] (Fig. 3).

The ERIC-PCR results for six *bla*_KPC_-positive *Enterobacter cloacae* isolates showed distinct profiles as follows: SL59 [80, 160, 280, 310, 350, 700, 950 bp] obtained from H01; isolate SL261 [310, 450 bp], recovered from H02; SL271 [310, 450 bp] isolated from H09; SL314 [280, 310, 500, 600, 750 bp], recovered from H11; and SL67 and SL75 [160, 280, 310, 450, 550 bp], which were recovered from H12 (Fig. 3).

Among isolates positive and negative for the *bla*_KPC_ gene, ERIC-PCR amplicons ranged between 80 and 900 bp for nine isolates identified as *Enterobacter aerogenes*. Two different profiles were observed for three strains negative for this gene. For the SL147 and SL193 isolates, bands of 80 and 160 bp were observed, and for SL80, the profile was 100, 280, 310, 450, 550, and 900 bp. The presence of *bla*_KPC_ was detected in six other isolates that showed heterogeneous patterns as follows: SL63 [160, 280, 450, 500 bp], recovered from H07; SL78 [160, 280, 310, 400, 500 bp], isolated from H09; isolates SL204 and SL205 [310, 450 bp] and SL211 [160, 280 bp], obtained from H05; and SL323 [160, 310, 500 bp], isolated from H12 (Fig. 3).

For five isolates identified as *Serratia marcescens*, ERIC-PCR fragments ranged between 80 and 310 bp. Isolates SL55, SL56 and SL209, which were negative for the *bla*_KPC_ gene, exhibited a single profile of 80 and 160 bp. The isolates SL04 and SL111, positive for *bla*_KPC_, showed bands of 80, 160, and 310 bp. These strains were isolated from patients treated at H05, a public hospital emergency room (Fig. 3).

The ERIC-PCR profiles for the three isolates identified as *Pantoea* spp. were 80, 200, 280, 400, and 650 bp for SL57 and 80, 160, 280, 350, 650, and 1200 bp for SL71. The strain SL207, which was positive for the *bla*_KPC_ gene and obtained from H15, showed a single fragment of 350 bp (Fig. 3).

Two strains identified as *E. coli* showed distinct ERIC-PCR profiles; *bla*_KPC_-positive SL141 was recovered from a patient at the H09 hospital, and showed fragments of 80, 160, 280, 350, and 550 bp, and the *bla*_KPC_-negative SL322 isolate had fragments of 160 and 250 bp (Fig. 3).

ERIC-PCR patterns of 80 and 160 bp were observed for three different isolates as follows: for those identified as *Raoultella planticola,* which included SL36 obtained from H10 and SL196 from H01, and for SL110, which was identified as *Raoultella ornithinolytica* and recovered from H10. The *bla*_KPC_ gene was detected in all these isolates.

For three *Proteus mirabilis* isolates, SL26, SL310 and SL313, a unique profile of 160 bp was observed. For the *Aeromonas salmonicida* strain SL210*,* fragments of 80 and 160 bp were found. All these isolates were negative for the *bla*_KPC_ gene (Fig. 3).

The distribution of different *bla*_KPC_-positive species in hospitals was as follows: for H01, *K. pneumoniae, Enterobacter cloacae* and *Raoultella planticola;* for H02, only *Enterobacter cloacae;* for H05*, K. pneumoniae, Enterobacter aerogenes* and *Serratia marcescens;* for H07, only *Enterobacter aerogenes;* for H08, only *K. pneumoniae;* for H09, *K. pneumoniae, Enterobacter cloacae, Enterobacter aerogenes* and *E. coli*; for H10, *K. pneumoniae, Raoultella planticola* and *Raoultella ornithinolytica;* for H11, only *Enterobacter cloacae;* for H12, *K. pneumoniae* and *Enterobacter cloacae;* and for H15, only *Pantoea* spp. It is important to note that H09, a public emergency room, had the highest number of different bacterial species carrying the *bla*_KPC_ gene.

In summary, the results of ERIC-PCR for 120 isolates belonging to the *Enterobacteriaceae* family showed that 79 (65.8%) bacterial strains carried the *bla*_KPC_ gene. Among these isolates, 26 (33.0%) clones were genetically distinct and were circulating in most [10/16 (62.6%)] of the hospitals surveyed.

Among the 297 bacterial isolates, 177 (59.6%) were non-fermenting Gram-negative bacilli from the genera *Acinetobacter* (*n* = 129) and *Pseudomonas* (*n* = 48). The profiles produced by ERIC-PCR with DNA of these microorganisms were quite heterogeneous, with fragments ranging from 70 to 1500 bp. Of the 177 isolates, 128 (72.3%) were identified as *A. baumannii* and one A*. ursingii* (SL159). Of the 128 isolates of *A. baumannii,* the majority (107, 83.5%) was negative for the *bla*_KPC_ gene, and this gene was found in only 21 (16.5%) strains.

**Fig. 4:**
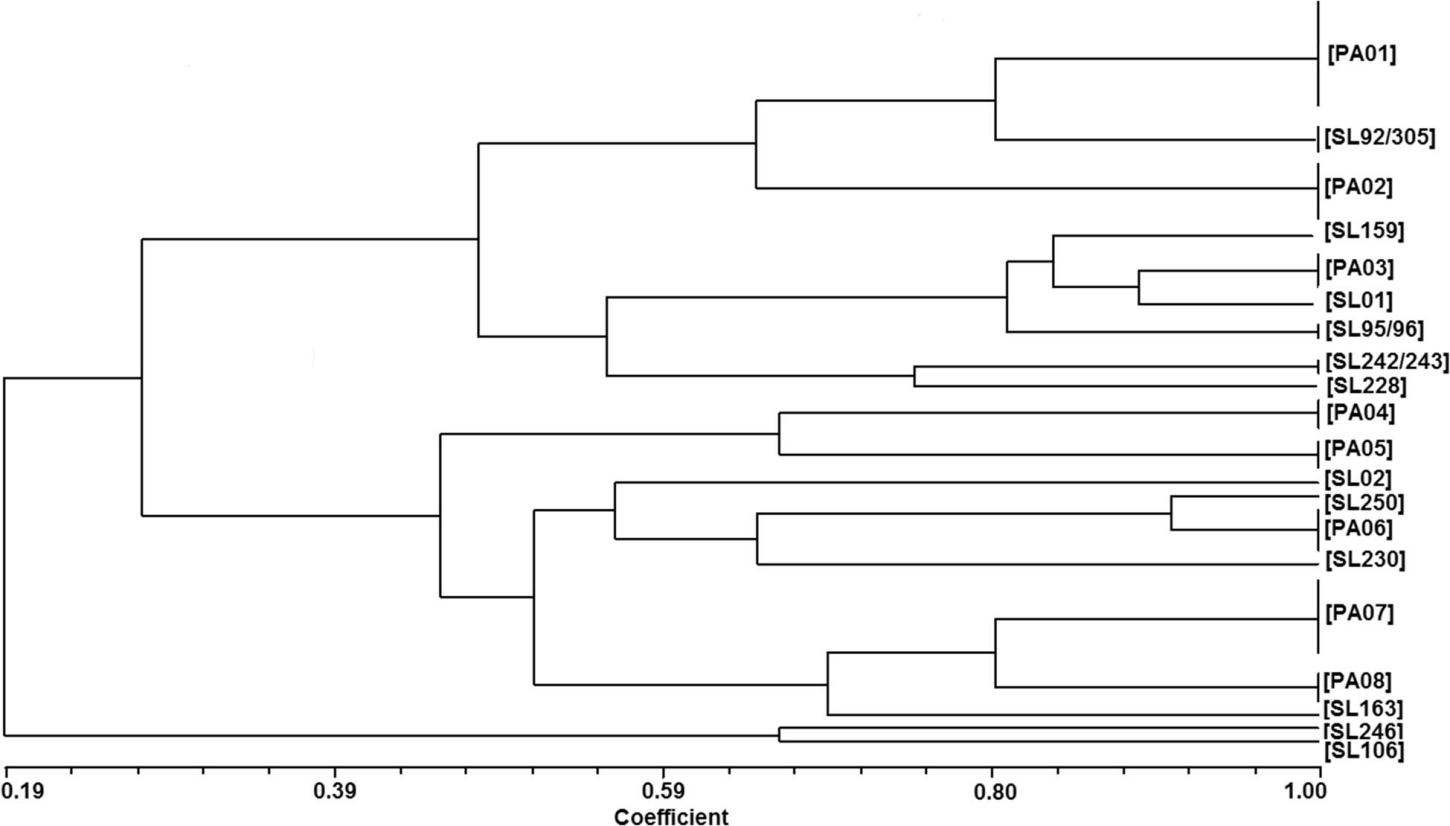
Dendrogram showing the genetic relatedness of 129 *Acinetobacter* spp. isolates. Cluster analysis of enterobacterial repetitive intergenic consensus PCR (ERIC-PCR) fingerprinting patterns using the DICE similarity coefficient and the UPGMA cluster method. The scale indicates the percentage of genetic similarity

The major genotyping profile found by ERIC-PCR for KPC-negative *A. baumannii* isolates was 100, 150, and 300 bp in 56 isolates (52.3%), which was named PA01 (Fig. 4), followed by 24 isolates with profiles of 100 and 150 bp (PA02 in Fig. 4). In addition, five isolates—SL90, SL91, SL97, SL124, and SL168—showed fragments of 100, 150, 300, 500, and 600 bp (PA03, Fig. 4); seven isolates—SL09, SL11, SL17, SL43, SL50, SL304, and SL306—had profiles with 100 and 350 bp (PA04, Fig. 4); and six isolates (SL84, SL87, SL93, SL99, SL101, SL135) had profiles of 100, 350, and 900 bp (PA05, Fig. 4). Heterogeneous patterns were observed for nine isolates, with fragments ranging from 100 to 1500 bp. Among these isolates, the profiles were as follows: SL95 and SL96 (150, 500, 600 bp); SL242 and SL243 (150, 300, 500, 900 bp); SL01 (150, 300, 480, 600, 900 bp); SL02 (150, 300, 350, 450, 900 bp); SL92 and SL305 (150, 300 bp); and SL228 (100, 150, 350, 500, 900, 1000, 1500 bp). The isolate SL159, identified as *A. ursingii*, was negative for the *bla*_KPC_ gene and showed the following profile: 150, 250, and 300 bp (Fig. 4).

ERIC-PCR analysis revealed that the 21 *bla*_KPC_-positive *A. baumannii* isolates had the following profiles: four isolates (SL231, SL232, SL233, SL235) with 100, 150, 350, 900, 1000, and 1500 bp, (profile PA06, Fig. 4); seven isolates (SL77, SL178, SL226, SL227, SL236, SL244, SL298) with 100, 150, 200, and 350 bp (PA07, Fig. 4); five isolates (SL100, SL107, SL125, SL126 and SL134) with 100, 150, and 350 bp (PA08, Fig. 4) and heterogeneous banding profiles for isolates SL106 [100, 150, 500, 900 bp]; SL163 [100, 150, 200, 250, 350, 750 bp]; SL230 [150, 350, 750, 1500 bp]; SL246 [100, 350, 500, 600, 750 bp]; and SL250 [100, 150, 200, 300, 500, 900 bp], as shown in Fig. 4.

Regarding the presence of *bla*_KPC_ in bacterial isolates, PCR assays revealed that of the 37 strains of *A. baumannii* obtained from H01, this gene was detected in seven (19%) of the clinical isolates (SL77, SL125, SL178, SL226, SL232, SL236, SL298), showing that three different bacterial clones containing the *bla*_KPC_ gene were circulating in H01 (Fig. 4, P06, PA07, PA08). In H02, [5/15 (33.3%)] five isolates—SL233, SL235, SL227, SL126 and SL106—had ERIC-PCR patterns very heterogeneous (Fig. 4, P06, PA07, PA08), showing that four different clones were circulating in this hospital. For H03, [1/6 (16.6%)] isolate SL246 showed a single profile. In H10, [8/44 (18.1%)] eight isolates were grouped into distinct profiles, including SL231 (PA06, Fig. 4); SL244 (PA07, Fig. 4); SL100, SL07 and SL134 (PA08, Fig. 4); and SL163, SL230, and SL250, which had heterogeneous profiles (Fig. 4), showing that six clones were circulating in this hospital.

**Fig. 5:**
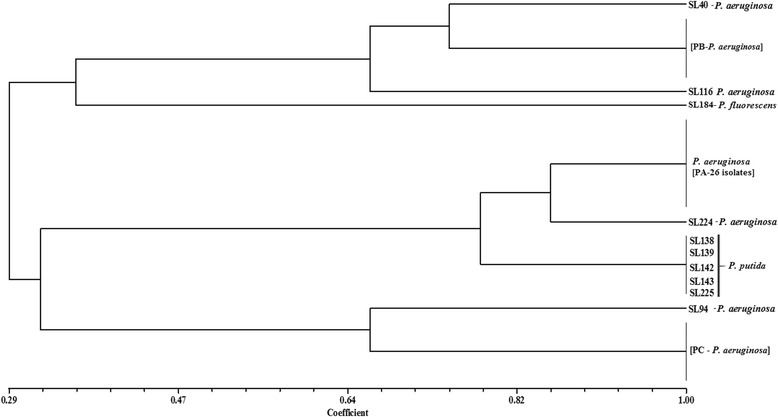
Dendrogram showing the genetic relatedness of *Pseudomonas* spp. isolates. Cluster analysis of enterobacterial repetitive intergenic consensus PCR (ERIC-PCR) fingerprinting patterns using the DICE similarity coefficient and the UPGMA cluster method. The scale indicates the percentage of genetic similarity

ERIC-PCR profiles for *Pseudomonas* spp. isolates showed different size fragments for the three different species. For *P. fluorescens* isolate SL184, the observed bands were 150, 200, and 250 bp; the five isolates (SL138, SL139, SL142, SL143 and SL225) identified as *P. putida* had a single profile of 100 and 150 bp. For the 42 *P. aeruginosa* isolates, the majority (26, 62%) showed the same profile, with fragments of 100, 150, and 300 bp (PA, Fig. 5); seven isolates (SL254, SL255, SL284, SL285, SL286, SL289, SL290) had bands of 150, 280, 320, and 600 bp (PB, Fig. 5); and for five isolates (SL256, SL257, SL258, SL259, SL293), the fragments were 100, 200,320, and 400 bp (PC, Fig. 5). Furthermore, four isolates showed heterogeneous profiles: SL40 [150, 250, 280, 320 bp]; SL94 [100, 200, 250, 320, 400 bp]; SL116 [150, 200, 280, 320, 1200 bp], and SL224 [100, 150, 300, 1000 bp]. It is important to note that the *bla*_KPC_ gene was not detected in bacilli belonging to the genus *Pseudomonas.*

## Discussion

This study showed the spread of multi-drug-resistant (MDR) bacterial strains isolated from clinical samples from patients in different healthcare facilities (public and private hospitals in northeastern Brazil) who showed resistance or decreased carbapenem antimicrobial susceptibility to imipenem, meropenem or ertapenem. The increasing prevalence of the clinical MDR-KPC phenotype has been associated with higher mortality rates, thereby posing a considerable threat to public health [21].

Our analysis indicated that *A. baumannii*, *K. pneumoniae* and *P. aeruginosa* species were the most frequently observed in clinical samples, with carbapenem resistance frequencies of 100, 84 and 100%, respectively. The high percentages of bacterial resistance to carbapenems and the cross-resistance observed to various antimicrobials are of concern in clinical medicine, especially in intensive care units [22]. It is possible that the genes encoding carbapenemases are located on genetic elements, such as integrons and transposons, in association with conjugative plasmids typically carrying genes for resistance to other antimicrobials [23]. Recently, the *bla*_KPC-3_ *, bla*_VIM-1_, *bla*_SHV-12*,*_ *bla*_OXA-9_ and *bla*_CMY-2_ genes were detected in a unique clinical multidrug-resistant *E. coli* isolate (clone ST448) carrying a *Tn4401* transposon associated with an IncFII plasmid [24]. Moreover, *bla*_KPC_ variant genes are typically located in *Tn4401* transposable elements and their isoforms, supporting their dispersion [25].

We found that several isolates of the *Enterobacteriaceae* family were resistant to most of the antimicrobials tested, except for Polymixin and amikacin. We observed that 10.7% of the *K. pneumoniae* strains were resistant to Polymixin B, with an MIC of 64 μg/ml. The other species were sensitive to Polymixin B. This antimicrobial drug is a good treatment option for *A. baumannii*, *E. aerogenes*, *E. cloacae* and *E. coli*, followed by amikacin, even in the presence of varying percentages of resistance [26].

This study has thus far been categorical in stating that the antimicrobial agent amikacin is a therapeutic option for the treatment of infections. Nevertheless, it has emerged, alone or in combination, as a major drug of interest for treating isolates that possess intrinsic resistance to Polymixin [26]. The combination of Polymixin with tigecycline has recently been described as a suitable option for the treatment of infections with MDR Gram-negative pathogens extended spectrum beta lactamase (ESBL- and carbapenemase-producing strains) [15]. However, increased expression of the efflux system in *A. baumannii* isolates has been correlated with a low level of susceptibility to tigecycline [27].

Molecular approaches aimed at detecting strains harboring carbapenemase genes are highly sensitive and efficient for confirmation of cases [28, 29]. Conversely, there are no methodologies to routinely conduct clinical and laboratory diagnoses [30]. In this respect, the presence of the *bla*_KPC_ gene must be confirmed by molecular biology techniques to define the production of KPCs [28]. The production of KPCs determined by the phenotypic modified Hodge test (MHT) showed a strong association with the presence of the *bla*_KPC_ gene by PCR (*p* < 0.0001). However, analyses showed that MHT positivity does not confirm the presence of KPCs, only the involvement of some KPCs that may or may not indicate a resistant phenotype. It is important to note that false positive results can occur when the MHT is used to detect carbapenemase in ESBL-producing isolates [7]. However, only one isolate was *bla*_KPC_ gene-positive and MHT-negative. Similar to our findings, patterns of antimicrobial resistance and prevalence of the *bla*_KPC_ gene were determined in *Enterobacteriaceae* species isolated from a hospital in India. Among 46 strains resistant to carbapenem antimicrobials, 38 (82.6%) tested positive by MHT, but the *bla*_KPC_ gene was detected in only 31 (67.4%) [31].

The emergence of antibiotic-resistant organisms is a major public health concern, particularly in hospitals and other health care settings, and represents a serious challenge for surveillance systems [32, 33]. Previous studies have shown the spread of KPC-type 2 and KPC-type 3 strains and have also identified new variants, such as bla_KPC-15_ [7, 34, 35]. Mortality rates of 18–72% have been reported in patients infected with bacteria carrying the *bla*_KPC_ gene in recent years [33, 36, 37], including those treated with combined therapy (tigecycline-gentamicin or tigecycline-colistin) or monotherapy using colistin or tigecycline for *Enterobacteriaceae* infections [15].

In Brazil, KPC-2 has been endemic since 2006 [10], and our surveillance systems have failed to detect KPC, its variants and their incidence rates. Moreover, we do not know the true prevalence of these carbapenemases and their impacts on hospital mortality rates. However, the few existing studies have suggested that the mortality rate associated with KPC infection in intensive care units in Brazil can reach 18% [38]. Recently, the first description of the *K. pneumoniae* clone ST258 associated with an outbreak in Ribeirão Preto city, Brazil was reported. This clone carried the transposon Tn4401a, which likely contributed to its spread [39].

Previous studies have indicated that the prevalence of the *bla*_KPC_ gene in *A. baumannii* is variable and not always indicative of β-lactam antimicrobial resistance with this type of KPC [40]. Other reports have indicated that high levels of resistance to carbapenems are strongly related to the presence of the *bla*_OXA-23_ and *bla*_OXA-24_ genes [41]. Furthermore, this study showed that 21 (16.4%) *Acinetobacter* strains harbored the *bla*_KPC_ gene among the 128 bacterial strains evaluated. A more recent study found that despite the apparent spread of the *bla*_KPC_ gene in *A. baumannii* isolates causing nosocomial outbreaks, additional types of carbapenemases are involved in the activity of this bacterial species and are associated (or not) with other non-enzymatic mechanisms, including changes in outer membrane protein (OMPs) efflux pumps and penicillin-binding proteins (PBP) [42].

Moreover, it is important to note that all isolates previously identified by biochemical tests as *Acinetobacter* spp were confirmed by PCR for identification at the species-level. Among the various genotypic methods used to identify isolates of the genus *Acinetobacter*, the PCR of the *bla*_*oxa51*_ gene has been proposed as a method to identify *A. baumannii* [19].

In *Enterobacteriaceae* carrying the *bla*_KPC_ gene, a high level of sensitivity to Polymixin was observed; however, it is worrisome that some strains showing resistance to this antimicrobial agent have emerged, including pathogens with intrinsic resistance. Previous reports have described the presence of KPC-3-producing *K. pneumoniae* that are resistant to colistin (Polymixin E) in hospitals in Sicily, Italy [21, 43]. In addition, high percentages (83.1%) of ciprofloxacin resistance have been observed in *K. pneumoniae* strains carrying the *bla*_KPC_ gene*,* indicating that few antimicrobial options may be available because these microorganisms accumulate different mechanisms of resistance.

Molecular genotyping using ERIC-PCR fingerprinting showed heterogeneous profiles that could differentiate bacterial species that contained these repetitive elements. ERIC-PCR patterns showed 26 distinct clones for eight bacterial species of the family *Enterobacteriaceae* and eight different clones for strains of *Acinetobacter baumannii* that were resistant to carbapenems and that were circulating in 11/16 (68.7%) of the hospitals surveyed.

The high discriminatory ability of the ERIC-PCR technique shown here is relevant and similar to others studies [44–51].

## Conclusions

The results of this molecular epidemiology survey indicate that bacteria of the *Enterobacteriaceae* family are still common carriers of *bla*_KPC_ gene variant 2 (KPC-2). However, the large number of *Acinetobacter baumannii* isolates carrying KPC-2 and KPC-3 variants is worrisome because this microorganism is an important opportunistic MDR pathogen that is frequently found to cause infections in inpatients and individuals throughout the community. Here, we have demonstrated the importance of monitoring hospitalized patients for the further emergence of carbapenem resistance in these bacteria as well as other Gram-negative pathogens. The high rates of clones carrying the *bla*_*KPC*_ gene suggest the need to improve the quality of health care to reduce the incidence of infections.

## Abbreviations

AST: Antimicrobial susceptibility test
CLSI: Clinical and Laboratory Standards Institute
EDTA: Ethylenediamine tetraacetic acid
ERIC: Enterobacterial repetitive intergenic consensus
GN: Gram-negative
*KPC*: *Klebsiella pneumoniae* carbapenemase
MDR: Multidrug-resistance
MHT: Modified Hodge test
MIC: Minimum inhibitory concentration
OXA: Oxacilinases
PCR: Polymerase chain reaction
TAE: Tris-acetate EDTA
UV: Ultraviolet light
